# Supplementary material for: Magnetically Stimulated Myogenesis Recruits a CRY2-TRPC1 Photosensitive Signaling Axis
Source: Cells. 2025 Feb 6;14(3):231. doi: 10.3390/cells14030231 (PMC11817702; doi:10.3390/cells14030231)
Supplement: Supplementary file 1 [file cells-14-00231-s001.zip › cells-3457769-supplementary.pdf]

Supplementary Material

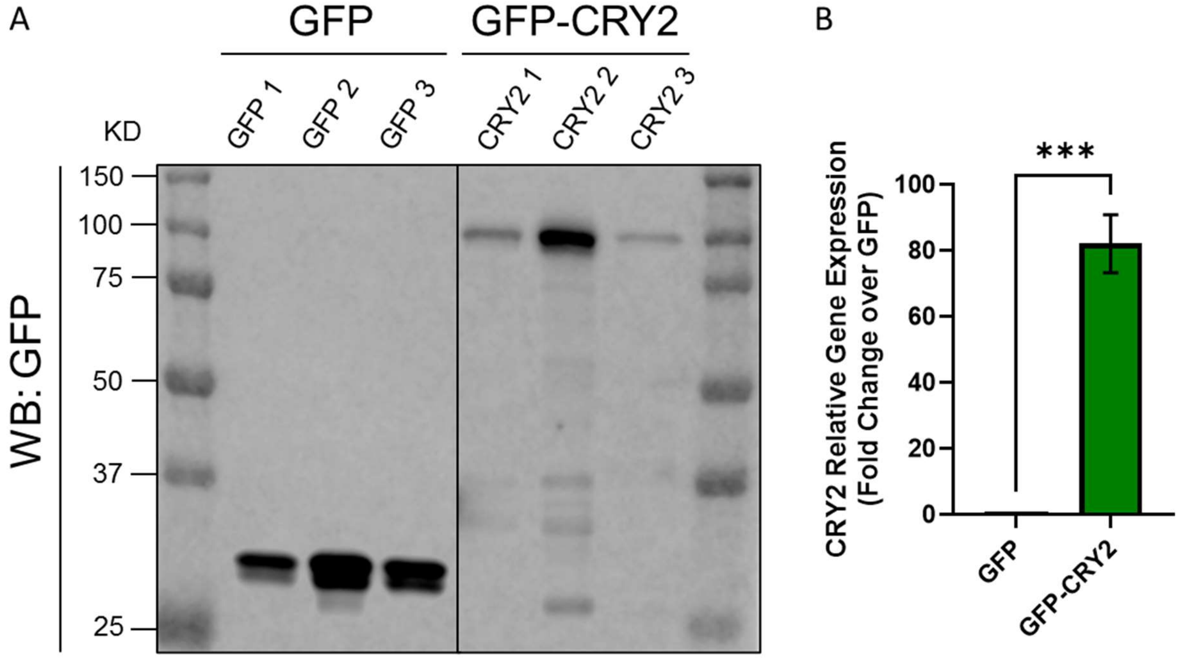

Supplementary Figure S1. Characterization of GFP-CRY2 overexpression in C2C12 cells. (A) Protein expression of GFP and GFP-CRY2 through western blot analysis. GFP protein was detected at 27 kDa and GFP-CRY2 at 82 kDa. Three single-sorted clones of GFP and GFP-CRY2 were compared, whereby CRY2-2 was subsequently used for experimental analysis due higher expression compared to the other two GFP-CRY2 clones. (B) Validation of CRY2 overexpression through qPCR analysis. GFP-CRY2 represents the CRY2-2 clone used previously, where it showed 82X CRY2 gene expression compared to GFP vector. Data represent three independent biological replicates, and statistical analysis was performed using unpaired t-test with *** $p < 0.001$. Error bars represent the standard error of the mean (SEM).

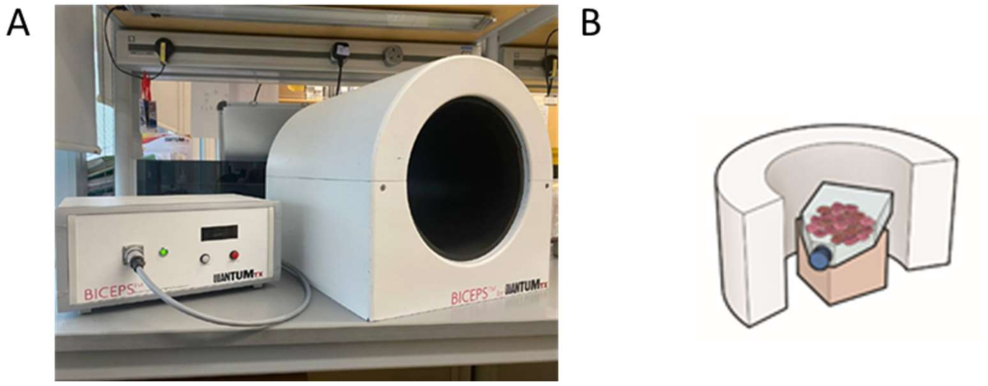

Supplementary Figure S2. (A) Pulsed electromagnetic field (PEMF) device connected to control box. (B) Cutaway view of device to allocate plated cells to be subjected to PEMF exposure.

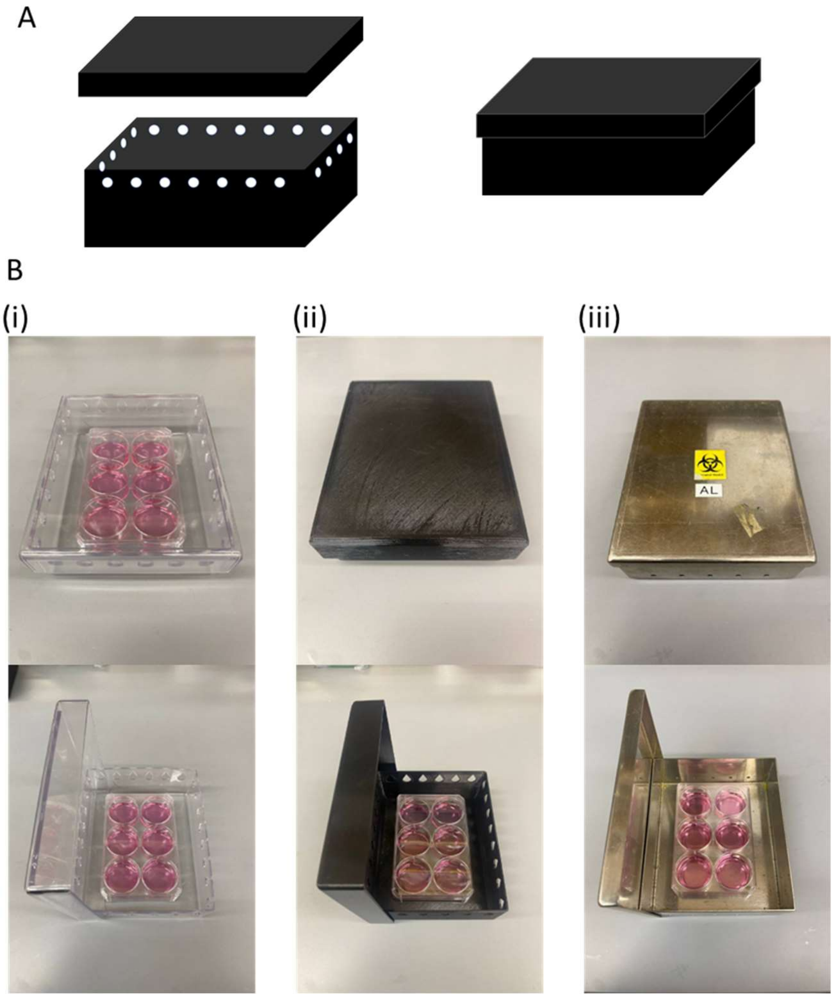

Supplementary Figure S3. (A) Schematic of black box fabricated using 3D printing. The design and dimensions of the black box were adapted from the µ-metal box with the adaptation that the gas exchange portals were placed further up the box and beneath the overhanging lip of the lid so as to minimize the entry of light. Clear box follows the dimensions of black box as a control. (B) Plated cells subjected to (i) light (clear box), (ii) dark (black box), and (iii) ambient field-shielding (µ-metal box).

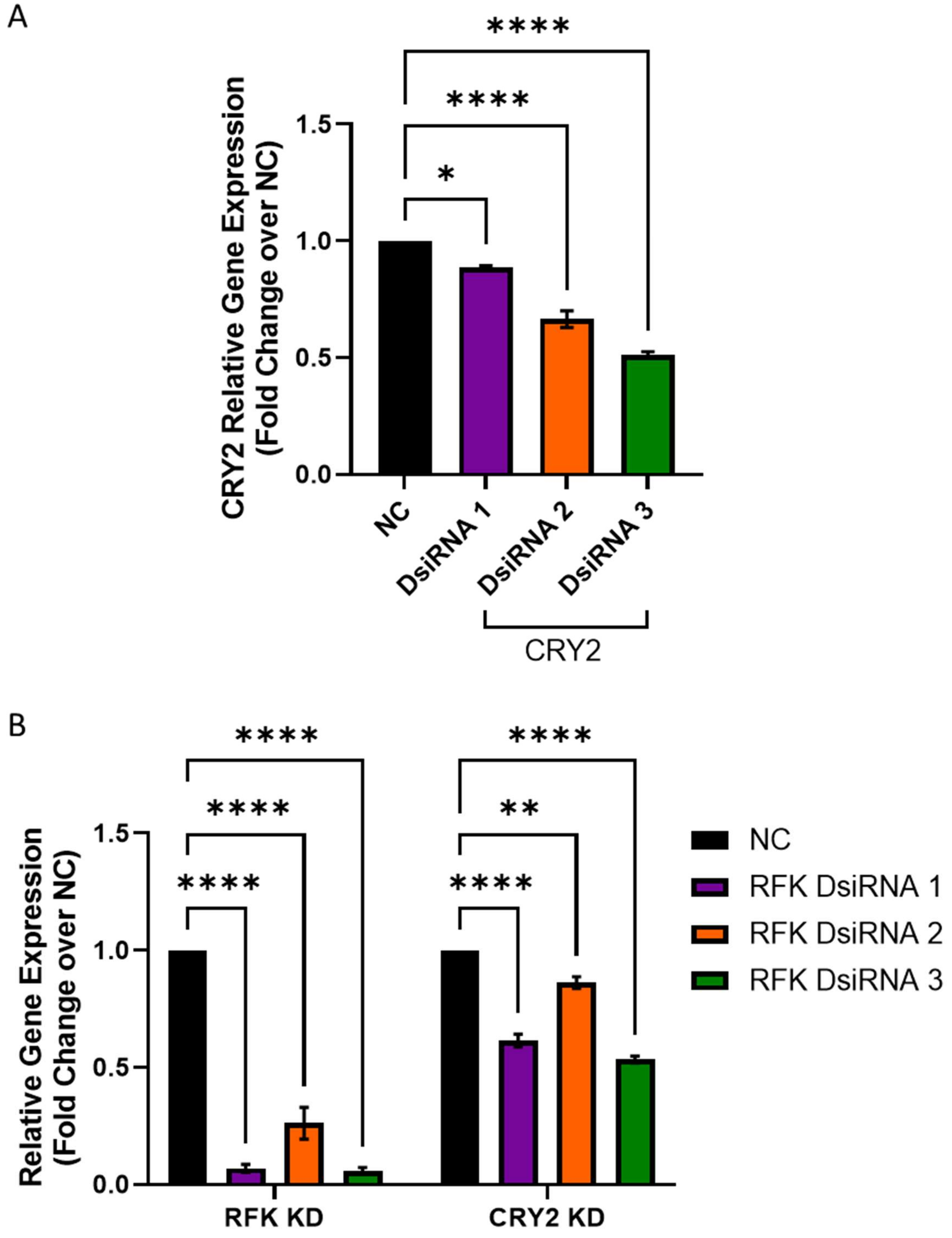

Supplementary Figure S4. Validation of CRY2 and RFK silencing through qPCR analysis. (A) One out of three pre-designed dicer-substrate short interfering RNAs (dsiRNA) was able to knockdown ~50% of the CRY2 gene, thus CRY2 dsiRNA 3 was used for experimental analyses. (B) Two out of three pre-designed dsiRNAs were able to knockdown ~95% of the RFK gene as compared to the scrambled/negative control, where RFK dsiRNA 3 was able to silence ~50% of the CRY2 gene and was thus used. Data represent three independent

biological replicates and statistical analysis was performed using one-way ANOVA followed by multiple comparison tests with * $p < 0.05$, ** $p < 0.01$ and **** $p < 0.0001$. Error bars represent the standard error of the mean (SEM).
